# Concurrent esophageal squamous cell carcinoma with gastric mucosa-associated lymphoid tissue lymphoma: a case report

**DOI:** 10.3389/fonc.2025.1582530

**Published:** 2025-10-17

**Authors:** Ting Xu, Shuai Luo

**Affiliations:** Department of Pathology, Affiliated Hospital of Zunyi Medical University, Zunyi, Guizhou, China

**Keywords:** primary, esophagus, squamous cell carcinoma, stomach, MALT lymphoma, combined

## Abstract

**Background:**

Double primary malignant tumors are infrequently encountered, with esophageal squamous cell carcinoma (ESCC) co-occurring with malignancies in other organs, representing a rare clinical entity. The concomitant presence of ESCC and lymphoma is even more uncommon, posing substantial diagnostic and therapeutic challenges.

**Case demonstration:**

A 76-year-old Han Chinese man presented with dizziness, dysphagia, and vomiting. Computed tomography suggested a potential esophageal neoplasm. Gastroscopy identified an ulcerated esophageal mass, accompanied by altered gastric body morphology and branching changes. Histopathological examination of biopsied esophageal and gastric mucosa confirmed ESCC concurrently with gastric mucosa-associated lymphoid tissue (MALT) lymphoma. The patient declined surgical intervention and succumbed 13 months after diagnosis.

**Conclusions:**

The simultaneous occurrence of ESCC and gastric MALT lymphoma remains exceedingly rare. In ESCC cases, the potential for coexisting malignancies in the pharynx, stomach, or other sites warrants consideration. This report documents the first known case of ESCC coexisting with gastric MALT lymphoma, contributing to the current understanding of diagnosis and management strategies in lymphoma-associated ESCC cases.

## Background

Double primary malignant tumor (DPMT) denotes the presence of two distinct malignant neoplasms occurring either within the same organ/system or across separate ones. The incidence of DPMT has markedly increased in recent years (1), with esophageal squamous cell carcinoma (ESCC) frequently coexisting with primary malignancies in other organs. Reported incidence rates range from 9.5% to 21.9% (2, 3). The most prominent secondary primary malignancies associated with ESCC are head and neck cancer (49.5%), lung cancer (40.2%), and gastric cancer (5.5%) (4). In contrast, secondary primary lymphoma remains exceedingly rare, with only a few cases documented to date (5, 6). This report delineates an exceptionally rare presentation of synchronous ESCC and gastric mucosa-associated lymphoid tissue (MALT) lymphoma, thereby enriching the current understanding and informing clinical management strategies for ESCC with rare lymphomatous comorbidities.

## Case demonstration

**Figure 1:**
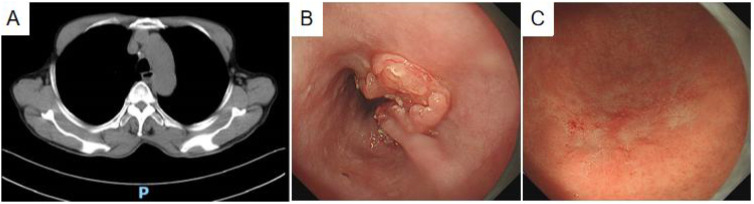
Diagnostic imaging. **(A)** Plain thin-slice CT scan: uneven wall thickening of the mid-thoracic esophagus with narrowing of the lumen. **(B)** Painless gastroscopy: the esophagus showed an ulcerated mass with white fur at the base, raised surrounding mucosa, and stiff plica. **(C)** A discolored tone change was seen in the greater curvature of the lower part of the gastric body, showing a dendritic change.

A 76-year-old Han Chinese man presented with a 6-month history of dizziness and a 10-day history of dysphagia accompanied by vomiting. Upon admission to the emergency department, the patient was afebrile without cough or sputum production. Physical examination revealed a conscious state, soft abdomen with localized and rebound tenderness, absence of renal percussion pain, and no apparent limb deformities. Muscle strength and tone remained within normal limits, and no lower limb edema was identified. Chest computed tomography (CT) (**Figure 1A**), including thin-layer flat scan and 3D reconstruction, demonstrated uneven thickening of the mid-esophageal wall with luminal narrowing and proximal esophageal dilatation, suggestive of esophageal carcinoma. Additional findings included radiologic features of chronic bronchitis and emphysema, mild pneumonia in the left lower lobe, and pulmonary calcifications scattered bilaterally. Multiple calcified lymph nodes were also noted in the mediastinum and bilateral hila.

The patient presenting with dysphagia and vomiting underwent an emergency chest CT and 3D evaluation, revealing esophageal cancer. Following symptomatic management, referral to the outpatient digestive clinic was advised by emergency department physicians. Painless gastroscopy subsequently identified an ulcerated esophageal mass located 25–36 cm from the incisors, featuring a white-coated base (**Figure 1B**). The surrounding mucosa appeared bulging, wrinkled, and stiff, while Magnifying Narrow Band Imaging (M-NBI) Intraepithelial Papillary Capillary Loop (IPCL) demonstrated B3 alterations. No irregularities in mucosal shape or color were detected. At 40 cm from the incisors, the cardia exhibited normal motility and a distinct dentate line. Although the gastric mucosa was largely unremarkable, the greater curvature of the lower gastric body displayed tone fading (**Figure 1C**), dendritic vascular changes, a positive demarcation line (DL), and dilated, twisted microvessels in the absence of overt mucosal or morphological abnormalities. The gastric angle appeared curved, with coarse mucosal surfaces showing redness and congestion, yet no brown pigmentation, extending into the middle gastric curvature. The pylorus was round and functionally intact. Differential diagnoses based on endoscopic findings included 1) progressive esophageal cancer, 2) lymphoma of the lower gastric body, and 3) chronic atrophic gastritis (C-2). Biopsies were obtained from the esophageal ulcerated lesion and three sites along the greater curvature of the lower gastric body.

The pathological biopsy of the esophagus revealed a small specimen measuring 4 × 3 × 2 mm with a slightly brittle texture. In contrast, the gastric body biopsy comprised three grayish tissues measuring 8 × 5 × 4 mm, characterized by a medium to soft texture.

**Figure 2:**
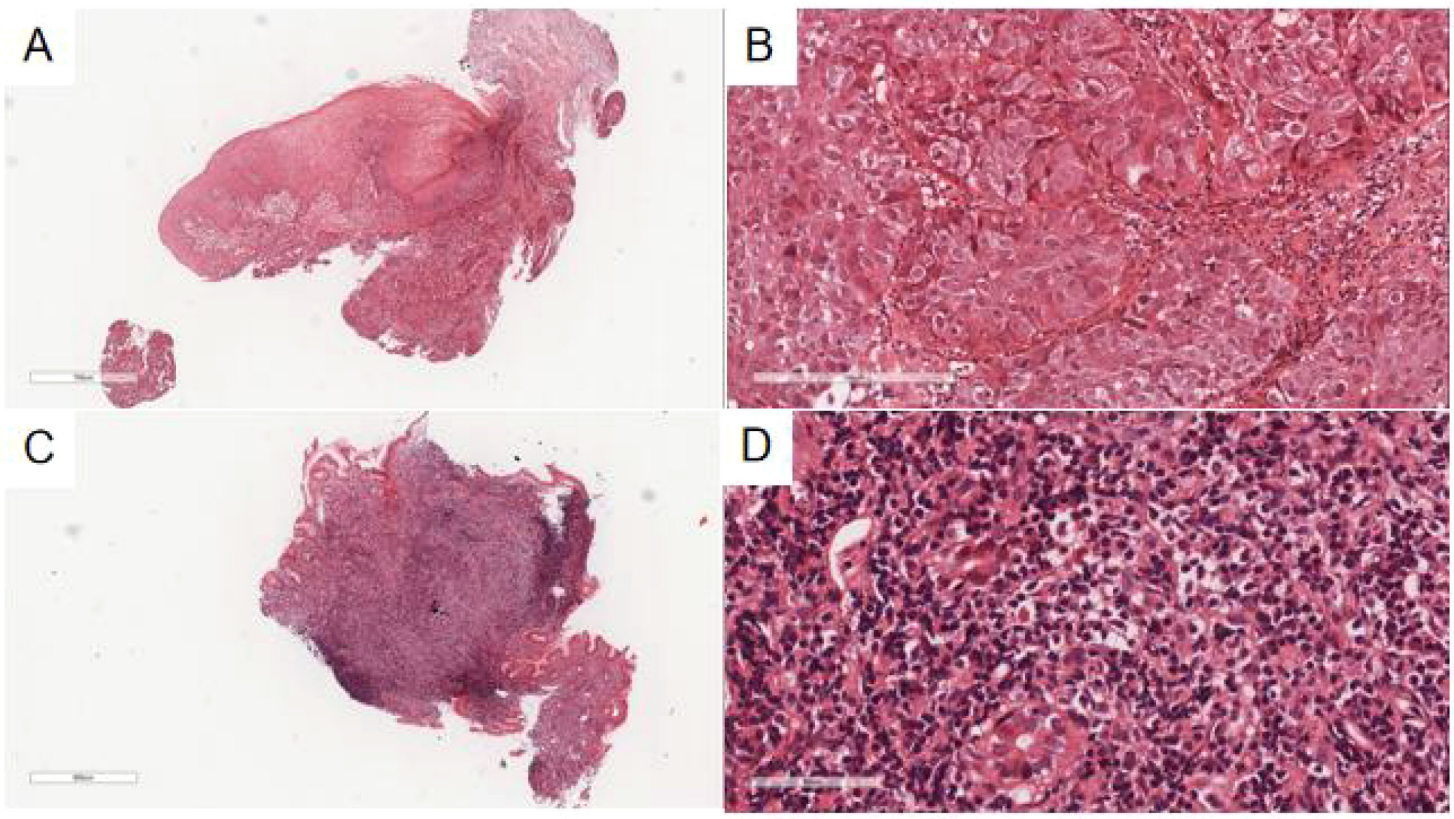
**(A)** The esophageal ulcer mass showed irregular nests of tumor cells under the normal squamous epithelium of the esophagus with invasive growth at low magnification. H&E, ×30. **(B)** The tumor cells were round and polygonal, with large, vacuolated nuclei and visible mitotic figures under high magnification. H&E, ×200. **(C)** At low magnification, the glands in the lamina propria of the gastric mucosa were reduced and replaced by diffuse patches of small lymphoid cells in the lesion area of the gastric body. H&E, ×26. **(D)** At high magnification, the lesion area of the gastric body was composed of different numbers of centrocyte-like cells, monocyte-like cells, and small lymphocyte-like cells, with slightly irregular nuclear outlines, heterogeneous sizes, and rare mitotic figures. H&E, ×400.

The esophageal ulcerated mass biopsy exhibited normal squamous epithelium at low magnification (**Figure 2A**), beneath which irregular, nest-like clusters of tumor cells infiltrated the submucosal muscle layer. At high magnification (**Figure 2B**), the tumor cells displayed diverse morphologies—round, polygonal, and spindle-shaped—with well-demarcated borders, enlarged nuclei, vacuolization, focal hyperchromasia, prominent nucleoli, and evident mitotic figures. Scattered inflammatory and basal cells surrounded the tumor nests, while keratinized beads were not observed.

The gastric mucosa biopsy (**Figures 2C, D**) demonstrated a marked reduction or complete absence of glands within the lamina propria, replaced by small lymphoid cells. In select regions, the glandular structure remained preserved, yet was densely infiltrated by numerous small lymphoid cells dispersed throughout the stroma. Residual glandular epithelium exhibited infiltration by neoplastic lymphocytes, forming lymphoepithelial lesions, defined by the aggregation of three or more neoplastic lymphocytes within mucosal glands. Under high magnification, tumor cells appeared heterogeneous, comprising central cell-like, monocyte-like, and small lymphocyte-like morphologies, with mildly irregular nuclear contours, variable sizes, and infrequent mitotic figures. Scattered eosinophils were also present.

**Figure 3:**
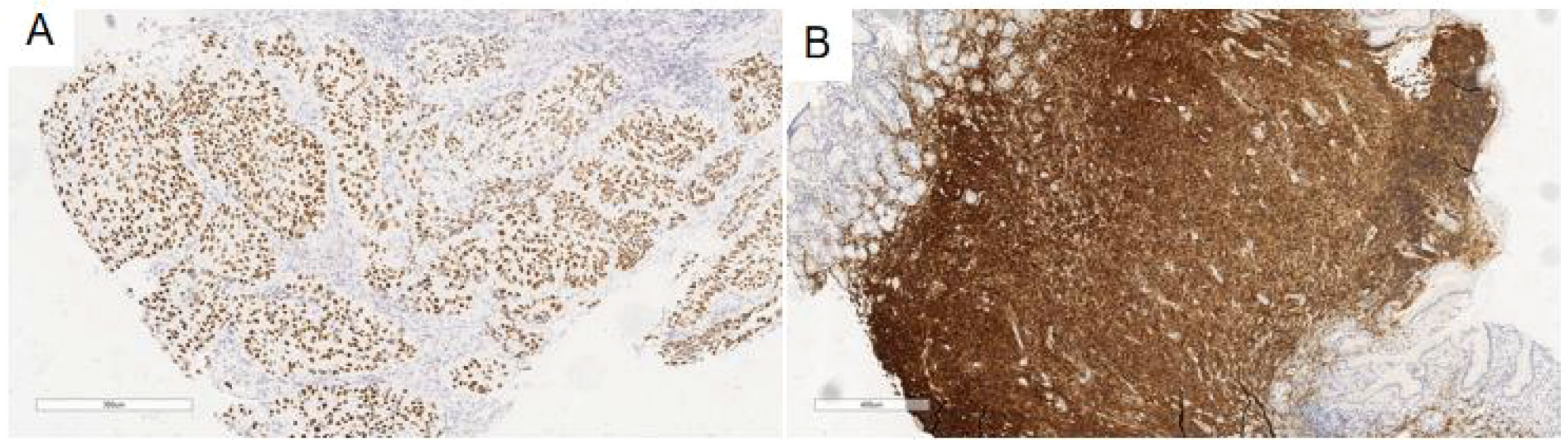
**(A)** Tumor cells at the esophageal ulcer mass were P40(+). EnVision, ×100. **(B)** Small lymphoid cells in the gastric body were CD20(+++). EnVision, ×50.

Immunohistochemical analysis revealed that tumor cells in the esophageal ulcer mass were positive for CK, P40, and P63 (**Figure 3A**) and negative for CAM 5.2 and CK7 and exhibited a Ki-67 labeling index of 80%.

In the gastric body region with color alteration, small lymphoid cells demonstrated strong LCA and CD20 positivity (**Figure 3B**) and were positive for BCL-2. CD43 showed partial positivity, while CD21 revealed a sparse, fragmented follicular dendritic network with focal Follicular Dendritic Cell(FDC) expression. Lymphoid cells were negative for CK, contrasting with the CK-positive glandular epithelium, indicating the presence of lymphoepithelial lesions. Additionally, CD3, CD5, CD23, Cyclin D1, SOX-11, and TdT were all negative, and no light chain restriction for Kappa or Lambda was detected. The absence of CD10 expression suggested a lack of germinal center formation within the lymphoid dendritic network, supporting the presence of follicular implantation. Ki-67 labeling index was 10%.

**Figure 4:**
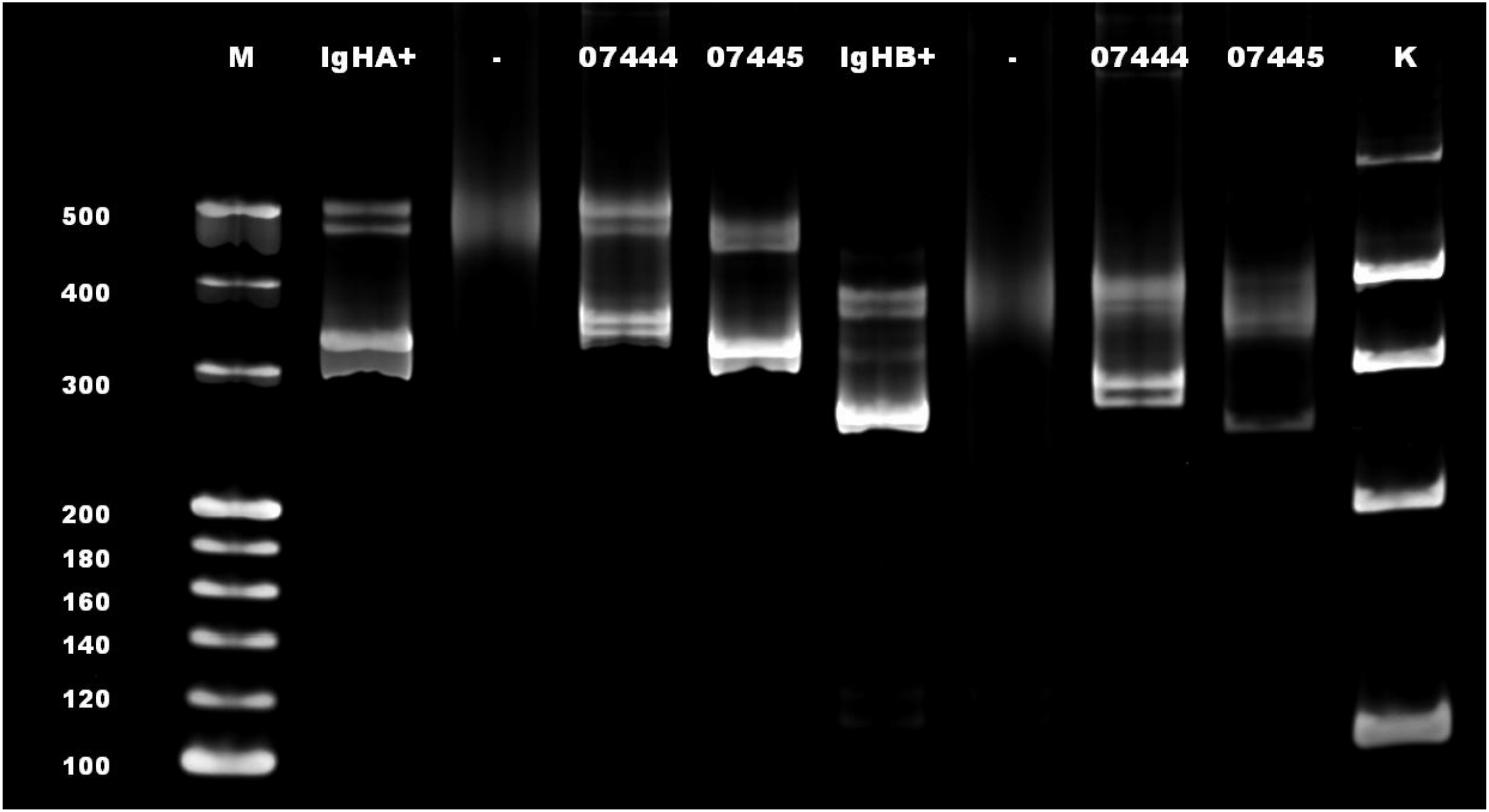
A rearrangement band was observed at 250–295 bp in IgHB. M, mark; IgHA+, positive control for IgHA; IgHB+, positive control for IgHB; -, blank control; 07444 and 07445, sample test number (07445 in this case); K, mark of the internal parameters.

Molecular pathology identified clonal rearrangements of B-cell IgH (**Figure 4**), Igκ, and Igλ genes, with recombinant bands observed at 310–360 bp for IgHA, 250–295 bp for IgHB, 120–160 bp for IgκA, 270–300 bp for IgκB, and 135–170 bp for Igλ. No distinct amplification bands were detected within the target fragment ranges for IgHC, IgHD, or IgHE.

Integrating endoscopic observations, histopathological features, and immunohistochemical profiles, a diagnosis of synchronous double primary ESCC with gastric MALT lymphoma was established.

The patient, who did not receive surgical intervention, succumbed 13 months after diagnosis due to progressive respiratory compromise and malnutrition.

## Discussion

DPMTs are rare neoplasms characterized by the following criteria: 1) each tumor is histopathologically confirmed as malignant; 2) the tumors arise in distinct anatomical sites or organs, separated by normal tissue; and 3) metastasis or recurrence is definitively excluded. Based on the diagnostic interval, DPMTs are categorized as either simultaneous or metachronous. Simultaneous DPMTs involve diagnoses of both malignancies within 6 months, while metachronous DPMTs are identified more than 6 months apart. Among ESCC patients with a second primary malignancy, hypopharyngeal carcinoma is most frequently observed, followed by oropharyngeal and laryngeal carcinomas (6). The stomach represents the second most common site for multiple primary tumors associated with esophageal cancer, with adenocarcinoma being predominant and lymphoma remaining exceptionally rare (approximately 0.6%) (7). This report delineates an exceptionally rare case of ESCC coexisting with gastric MALT lymphoma, intended to enhance current understanding of its diagnostic and therapeutic considerations.

Clinicians must sustain a heightened level of vigilance for DPMTs in clinical settings, particularly when patients exhibit pronounced symptoms or mass lesions suggestive of a potential second primary tumor. In this case, the patient reported a 6-month history of dizziness and dysphagia accompanied by vomiting. CT imaging identified esophageal cancer. During gastroscopy, the endoscopist meticulously evaluated the gastric mucosa following esophageal stent placement and performed a biopsy to investigate additional lesions. Although gastric mucosa-associated lymphoid lymphoma is exceedingly rare, the possibility of secondary malignancies in patients with ESCC warrants consideration. Beyond the frequently observed association between ESCC and second primary tumors in the oropharynx and hypopharynx, attention should also focus on the stomach and other anatomical regions for potential neoplastic involvement.

The pathogenesis of esophageal cancer stems from a multifactorial interplay of genetic, environmental, and lifestyle influences, including smoking, intake of overheated or coarse foods, nutritional deficiencies, and inadequate oral hygiene (8). In contrast, MALT lymphoma is predominantly associated with chronic infections or autoimmune conditions. Approximately 90% of patients with gastric MALT lymphoma test positive for *Helicobacter pylori* (9). Eradication of *H. pylori* remains the first-line treatment, with lesion regression or clinical remission observed in nearly 75% of cases (10). Clinically, this patient exhibited dysphagia and vomiting caused by marked esophageal obstruction, while other gastrointestinal symptoms—such as abdominal distension, anorexia, acid reflux, or belching—were absent. Consequently, the gastric lesions may have been overlooked due to the prominence of the esophageal pathology. Nevertheless, a comprehensive evaluation of the gastric mucosa was performed, and biopsies were obtained from the affected regions.

The diagnosis of ESCC remains straightforward due to its distinct histomorphological features and characteristic immunohistochemical markers, including P40, P63, and CK5/6 (11). In contrast, diagnosing gastric extranodal MALT lymphoma necessitates a more exhaustive assessment integrating site-specific, morphological, immunohistochemical, and molecular parameters (12). Histopathologically, a diffuse infiltration of small lymphocytes in the mucosa and lamina propria was observed, exhibiting mild nuclear atypia. Immunohistochemistry demonstrated positivity for LCA, CD20, BCL-2, and CD43, while CK, CD20, CD5 (−), and CD23 (−) findings supported the exclusion of small cell lymphoma. The absence of CyclinD1 (−) and SOX-11 (−) ruled out mantle cell lymphoma, and TdT (−) excluded lymphoblastic lymphoma. Moreover, unrestricted expression of Kappa and Lambda chains ruled out plasmacytoma, while CD10 (−) and BCL-6 (−) findings suggested the exclusion of follicular lymphoma. Given that MALT lymphoma is typically low-grade, Ki-67 expression remained minimal. Monoclonal B-cell rearrangement was confirmed via molecular pathology (13). The diagnosis in this case was substantiated by a comprehensive integration of histomorphological, immunohistochemical, and molecular evidence.

Surgery remains the primary modality for early-stage ESCC, except when the lesion is restricted to the mucosal layer or when distant organ metastasis is evident, categorizing the disease as locally advanced ESCC. In cases of unresectable locally advanced ESCC, concurrent chemoradiotherapy constitutes the standard therapeutic strategy (14). Gastric MALT lymphoma demonstrates a strong association with *H. pylori* infection, with eradication therapy regarded as the first-line intervention. Nonetheless, a substantial proportion of patients with gastric MALT lymphoma lack *H. pylori* infection, and the pathogenesis, endoscopic characteristics, and therapeutic approaches in these cases diverge markedly from those in infected individuals, thereby rendering the treatment paradigm controversial (15).

## Conclusion

The co-occurrence of multiple primary malignancies in other organs and ESCC remains relatively common in clinical practice. However, the synchronous presentation of ESCC and gastric MALT lymphoma is exceedingly rare. A heightened index of suspicion for secondary primary tumors remains crucial in such contexts. In addition to routine surveillance for ESCC, vigilance must also extend to adjacent anatomical sites, including the throat and stomach, to facilitate the timely detection of concomitant malignancies. This report delves into the first documented case of concurrent ESCC and gastric MALT lymphoma, highlighting its crucial value in refining diagnostic strategies and therapeutic decision-making for ESCC complicated by rare lymphoid neoplasms.

## Data Availability

The original contributions presented in the study are included in the article/supplementary material. Further inquiries can be directed to the corresponding authors.
